# Performance analysis of neural network architectures for time series forecasting: A comparative study of RNN, LSTM, GRU, and hybrid models

**DOI:** 10.1016/j.mex.2025.103462

**Published:** 2025-07-08

**Authors:** Ariana Yunita, MHD Iqbal Pratama, Muhammad Zaki Almuzakki, Hani Ramadhan, Emelia Akashah P. Akhir, Andi Besse Firdausiah Mansur, Ahmad Hoirul Basori

**Affiliations:** a Department of Computer Science, Universitas Pertamina, Jl. Teuku Nyak Arief, South Jakarta, Jakarta, 12220, Indonesia; b Center of Data Science and Automation, Universitas Pertamina, Jl. Teuku Nyak Arief, South Jakarta, Jakarta, 12220, Indonesia; c Independent Researcher, Junusstraat, Wageningen, Gerderland, 6701AX, The Netherlands; d Department of Computing, Universiti Teknologi Petronas, Bandar Seri Iskandar, Perak Darul Ridzuan, Perak, 32610, Malaysia; e Faculty of Computing and Information Technology in Rabigh, King Abdulaziz University, Rabigh, 21911, Makkah, Saudi Arabia

**Keywords:** Neural network, Recurrent neural network, Long short term memory, Gated-recurrent unit, Monte carlo simulation

## Abstract

Recurrent Neural Networks (RNNs), Long Short-Term Memory (LSTM) networks, and Gated Recurrent Units (GRUs) have gained significant popularity in time series forecasting across diverse domains including healthcare, astronomy, and engineering. However, the inherent variability in model performance due to random weight initialization raises questions about the reliability and consistency of these architectures for time series analysis. This study addresses this concern by conducting a comprehensive benchmark evaluation of nine neural network architectures: vanilla RNN, LSTM, GRU, and six hybrid configurations (RNN-LSTM, RNN-GRU, LSTM-RNN, GRU-RNN, LSTM-GRU, and GRU-LSTM). Performance evaluation was conducted using Monte Carlo simulation with 100 iterations across three real-world datasets: sunspot activity, Indonesian COVID-19 cases, and dissolved oxygen concentration measurements. Statistical analysis employed the Friedman test to assess performance differences across architectures. Results showed no statistically significant differences among the nine architectures. Despite the lack of statistical significance, consistent performance patterns emerged favoring LSTM-based hybrid architectures. The LSTM-GRU and LSTM-RNN configurations demonstrated superior performance across multiple evaluation metrics, with LSTM-RNN excelling in sunspot and dissolved oxygen forecasting, while standalone LSTM showed optimal performance for COVID-19 prediction. These findings provide evidence-based guidance for architecture selection in time series forecasting applications, suggesting that while statistical equivalence exists among architectures, LSTM-based hybrids offer practical advantages in terms of consistency and robustness across diverse temporal patterns.

## Highlights

• A Monte Carlo method to assess machine learning time series algorithms is outlined.
• Nine 2-hidden-layer algorithms with RNN, LSTM, and GRU structures are evaluated. These are RNN, LSTM, GRU, RNN-LSTM, RNN-GRU, LSTM-RNN, GRU-RNN, LSTM-GRU, GRU-LSTM.
• Over a hundred iterations, LSTM performs the best on one time series dataset and LSTM-RNN on the other two datasets.
• Although no method is universally optimal, RNN is the fastest among all methods, and LSTM-RNN is generally faster than LSTM-GRU.

## Graphical abstract

**Figure:**
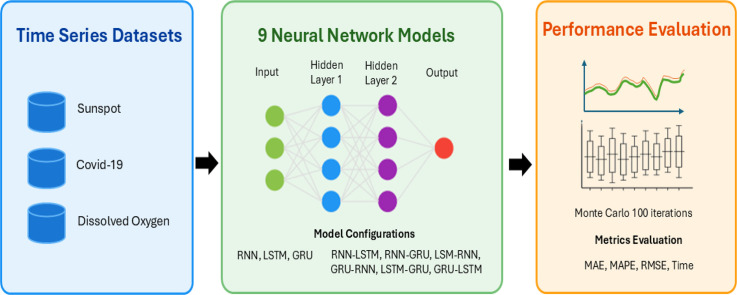

**Specifications table**

**Table:** 

**Subject Area**	Computer Science
**More Specific Subject Area**	Time Series Forecasting
**Name of The Reviewed Methodology**	Recurrent Neural Networks (RNN), Long-Short Term Memory (LSTM), Gated Recurrent Units (GRU), RNN-LSTM, LSTM-RNN, RNN-GRU, GRU-RNN, LSTM-GRU, GRU-LSTM
**Keywords**	Neural Network, Recurrent Neural Network, Long Short Term Memory, Gated-Recurrent Unit, Monte Carlo Simulation
**Resource Availability**	Sunspot Dataset- downloaded from https://www.kaggle.com/datasets/robervalt/sunspots ; Covid-19 Dataset- downloaded from https://www.kaggle.com/datasets/hendratno/covid19-indonesia ; Dissolved Oxygen Dataset (available from the corresponding author on reasonable request)
**Review Questions**	•How do different neural network architectures (RNN, LSTM, GRU and its hybrid models) with dual hidden layers perform across diverse time series datasets? •What are the comparative advantages and trade-offs between single and hybrid neural network architectures in terms of multiple performance metrics and computational efficiency for research and industrial applications? •To what extent does a hybrid model integrating LSTM, RNN, and GRU outperform single-algorithm approaches in terms of predictive accuracy and computational efficiency using Monte Carlo-based evaluation?

## Background

The rapid expansion of the internet and distributed sensor networks has led to a significant increase in the accumulation of time series data. This type of data, consisting of sequences indexed by time, is crucial in various fields such as finance, meteorology, inventory management, and health monitoring. Traditional approaches have primarily relied on parametric models guided by domain knowledge, including linear regression, moving averages, exponential smoothing, and ARIMA [1], [2]. In contrast, modern machine learning methods enable the learning of temporal dynamics purely through data-driven techniques. Previous studies have shown that deep learning architectures outperform traditional statistical methods [3], [4]. With the surge in data availability and computing power, machine learning has become an essential component of next-generation time series forecasting models. Time series data are essential for analyzing trends and recognizing patterns that change over time, thus enabling accurate forecasting.

A variety of deep learning architectures, such as recurrent neural networks (RNNs), long short-term memory (LSTM) networks, and gated recurrent units (GRUs), have been designed to address the diverse nature of time series datasets across various domains. Neural networks, which serve as the foundation for deep learning, use interconnected layers of nodes to learn and extract features from raw data, making them powerful tools for various machine learning applications. Several studies have proposed hybrid machine learning models incorporating multiple neural network approaches for forecasting time series data, such as RNN-LSTM [5], LSTM-RNN [6], [7], LSTM-GRU [8], and GRU-RNN [9].

These algorithms often rely on stochastic gradient descent or random initialization [10], which can result in varying results. Some studies propose a hybrid model, but the proposed models were trained only once. In particular, initial training may produce a poor-performing model, but retraining the same model may yield improved results. Hence, benchmarking these models to select models is crucial in specific time series analysis scenarios.

A benchmark study [11] demonstrated that ML techniques, including Neural Network-based ones, are reliable to estimate the scour depth of an experimental dataset with Monte Carlo (MC)-based evaluation. A Monte Carlo evaluation was applied on the performance of the ’non-linear autoregressive model process with eXogenous input’ (NARX), a RNN based algorithm with embedded memory, on time series data [12] and demonstrated that MC evaluation is more reliable than the traditional k-fold cross validation. Hence, we extend this approach to conduct a MC evaluation on the nine different neural network-based algorithm on time series data.

This study offers three key contributions: (1) a comprehensive benchmark of nine neural network architectures with dual hidden layers across diverse time series datasets, (2) implementation of Monte Carlo-based evaluation to guarantee the reliability of the benchmarked algorithm, and (3) comparative analysis of single and hybrid architectures based on multiple performance metrics and computational efficiency, providing practical guidelines for model selection in both research and industrial applications.

## Method details

### A review of RNN, LSTM and GRU for time series data

**Fig. 1:**
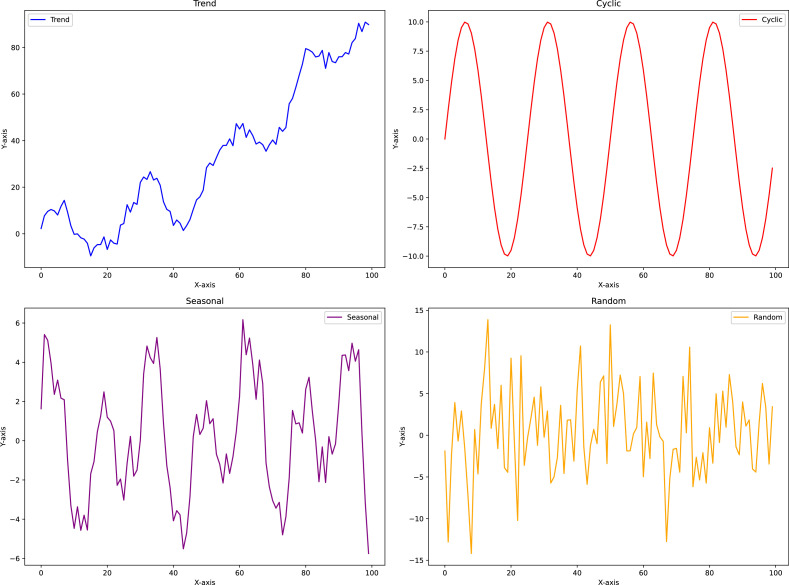
Examples of time series data patterns.

The main purpose of performing a time series analysis is to predict future values from existing historical data, which often show specific patterns such as trends, seasonal behavior, cycles, or random fluctuations, as illustrated in Fig. 1.

A trend is a sustained increase or decrease in the data values over an extended period. Trends can exhibit linear or nonlinear characteristics. An illustrative example is the persistent rise in global temperatures observed over the past several decades. A seasonal pattern repeats at consistent intervals as a result of seasonal influences. For example, retail sales typically increase annually during the holidays. A cyclical pattern consists of long-term fluctuations that lack the regularity of seasonal patterns and are frequently influenced by economic or business cycles. An example is the cyclical nature of stock markets. Finally, random patterns arise from unpredictable or random influences and do not adhere to any trend or seasonality. These patterns may result from sudden events, errors, or noise.

Neural networks have been widely applied in forecasting time series data. RNNs, LSTM networks, and GRUs are particularly useful for time series analysis, as they are capable of handling sequential data and learning long-term dependencies.

RNNs were developed to analyze time series data and have been used in various fields such as speech recognition, machine translation, and image captioning [13]. RNNs process the incoming time series data using a separate vector at each time step, keeping the information recorded at the previous time step hidden. The equations describing RNNs are as follows [14].

$$h_{t}^{R}=g\left(W\cdot x_{t}+U\cdot h_{t-1}^{R}+b\right)$$

$$O_{t}^{R}=g\left(W^{o}\cdot h_{t}^{R}+b^{o}\right)$$

Here, g is the activation function (either hyperbolic tangent or logistic), $O_{t}^{R}$ is the output/prediction, $x_{t}$ is the input at time t, $h_{t-1}^{R}$ is the previous hidden state, b is the bias, and W and U are weights.

The LSTM algorithm was developed by Hochreiter and Schmidhuber in 1997 [15]. LSTM is a subtype of the RNN model and addresses the issue of short-term memory in RNNs, in which prior information no longer influences the learning network. To this end, a cell state is added in the LSTM architecture, enabling the network to store past information in memory for a longer period [16].

An LSTM network contains a complex structure called an LSTM unit within the hidden layer, which comprises three gates: the input, forget, and output gates. These gates are leveraged to selectively add or retain information in the cell state, which functions to store a value or state.

GRUs are one of the most popular variants of RNNs. They feature a specialized neural network with dedicated gates, optimized based on LSTM. Their structure is similar to that of an LSTM network, but the key difference is that a GRU combines the input gate and forget gate into a single update gate. Thus, this model consists of two gates: the update gate, which controls how much of the previous information needs to be retained in the current state, and the reset gate, which determines whether the previous information and the current state should be connected [17].

The GRU is expressed using the following equations [18].

$$\begin{array}{cc} z_{t} & =\sigma (W_{xz}x_{t}+W_{hz}h_{t-1}+b_{z})\;\left(2.3\right)\\ r_{t} & =\sigma (W_{xr}x_{t}+W_{hr}h_{t-1}+b_{r})\;\left(2.4\right)\\ {\tilde{h}}_{t} & =\tanh(W_{xh}x_{t}+r_{t}⊙W_{hh}h_{t-1}+b_{h})\;\left(2.5\right)\\ h_{t} & =\left(1-z_{t}\right)⊙h_{t-1}+z_{t}⊙{\tilde{h}}_{t}\;\left(2.6\right) \end{array}$$

Here, $z_{t}$ is the update gate, $r_{t}$ is the reset gate, ${\tilde{h}}_{t}$ is the new candidate state, $h_{t}$ is the hidden state at time t, σ is the sigmoid function, ⊙ denotes element-wise multiplication, W represents the corresponding weights, and b is the bias.

### Related works about hybrid models

Wang et al. [5] proposed the RNN-LSTM network in the context of construction projects to predict and identify quality problems in steel bars, formworks, concrete, cast-in-place structures, and masonry that may occur during project implementation. These authors did not provide a comparison with other models.

Muhuri et al. [6] compared several classifier algorithms, namely, support vector machine (SVM), random forest (RF), and LSTM-RNN, using the NSL-KDD dataset for binary and multi-classification problems. For multi-classification, the LSTM-RNN network combined with a genetic algorithm achieved significantly higher accuracy than SVM and RF. In binary classification, the accuracy of LSTM-RNN was comparable to that of RF and exceeded that of SVM.

Another study by Pawar et al. [7] used several combinations of LSTM and RNN to forecast stock market prices. They compared LSTM-RNN with standalone LSTM and other architectures of LSTM and RNN. They proposed that the LSTM-RNN model provided more accurate results than traditional machine learning algorithms.

Zafar et al. [8] introduced an LSTM-GRU model to predict traffic speed in a specific city in Pakistan. The model integrated heterogeneous data from sensors, services, and external sources into a hybrid spatiotemporal feature space. They evaluated various models, including LSTM, GRU, CNN, LSTM-CNN, CNN-LSTM, GRU-LSTM, LSTM-GRU, CNN-GRU, and GRU-CNN, using three metrics: RMSE, MAE, and MAPE. Their findings revealed that the LSTM-GRU model outperformed all other approaches.

Xia et al. [9] used a stacked GRU-RNN model to predict renewable energy generation and determine the electrical loads required to support smart grid operations. Their experiments included two scenarios: forecasting wind power generation based on multiple weather parameters and predicting electrical loads using historical energy consumption data.

All related studies propose hybrid models based on RNN and its variations. However, the majority conduct only a single round of training and testing.

## Methods

**Fig. 2:**
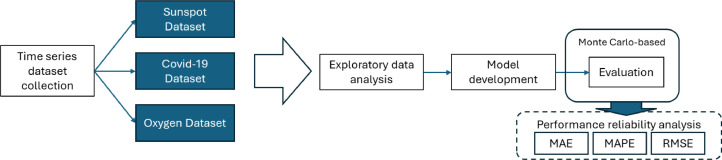
Overview of performance benchmarking over three datasets with the chosen algorithms.

The flow chart of our benchmarking approach to achieve the reliability of neural network-based models is depicted in Fig. 2. We collect three datasets in the domain of astronomy, healthcare, and machinery, respectively. Then, we conducted an exploratory data analysis (EDA) as a preliminary statistical and data quality analysis on the three datasets. After that, we develop the neural network-based models for the time series analysis. Finally, we evaluate the models with their random initialization in mind by repeating the experiments to gain sufficient data for reliability analysis.

### Datasets

We used three data sets in this study: 1) a sunspot dataset^2^, 2) a Covid-19 dataset [19]^3^, and 3) an oil and gas dataset of daily dissolved oxygen readings, as summarized in Table 1. The sunspot dataset contains sunspot data retrieved from the Kaggle website. Sunspots are transient phenomena observed in the Sun’s photosphere, manifesting as darker regions relative to the surrounding areas. These areas are characterized by a lower surface temperature, attributable to concentrations of magnetic flux that suppress convective processes. Typically, sunspots emerge in pairs with opposite magnetic polarity, and their occurrence fluctuates according to the approximately 11-year solar cycle. The dataset consists of the monthly mean total sunspot number observations, collected between 1749 and 2018. The COVID-19 dataset comprises the daily count of new COVID-19 cases in Jakarta, Indonesia, spanning 634 days from 1 March 2020 until 2 December 2021. The oxygen dataset consists of daily dissolved oxygen readings collected from an oil and gas plant.

**Table 1:** Summarized statistical and evaluation information of time series datasets.

	Datasets		
	Sunspot	Covid-19	Oxygen
**No. of Records**	3625	634	1,033
**Interval type**	Monthly	Daily	Daily
**Trend**	No	No	No
**Cyclic**	Yes	No	No
**Seasonal**	No	No	No
**Value range**	0–398	0–20.33	0–100
**Value mean**	82.64	10.45	37
**Value median**	68.3	10.97	32.39
**Value st. dev**	67.6	3.71	27
**Train-test ratio**	70:30	70:30	80:20

### Exploratory data analysis

**Fig. 3:**
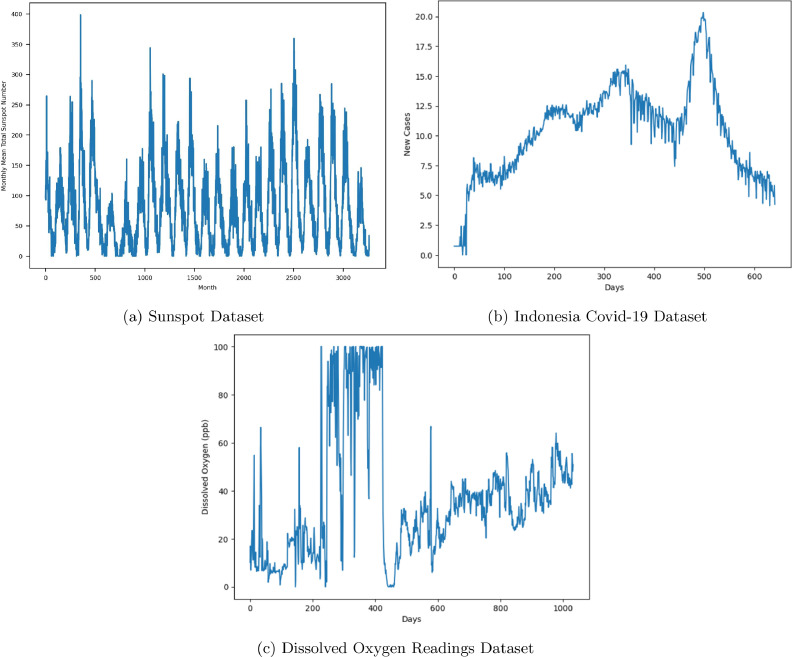
The visualization of the evaluation datasets for our benchmarking.

The EDA reveals that the datasets have distinct statistical information as summarized in Table 1. The sunspot, covid-19, and oxygen datasets has 3625, 634, and 1033 records, respectively, and do not have missing values. We transformed the Covid-19 dataset using boxcox because the data have a drastic change. As depicted in Fig. 3, the datasets may have outliers and can impact the performance analysis. However, we omit the outlier detection and removal because we want to observe the robustness of the benchmarked algorithms against the outliers.

The three datasets also show a time-dependent pattern, as depicted in Fig. 3, in which the value at any given time is affected by previous values in the time series. It remains unclear whether this dependency is influenced by external factors or is intrinsic to the temporal sequence itself. The discussion of time-dependent pattern is currently outside the scope of this research.

### Model development

As described in the previous section, hybrid models, such as GRU-LSTM are advantageous towards a sensor reading-based time series dataset. Thus, we benchmark both the original and hybrid methods of nine neural network-based machine learning algorithms, namely RNN, LSTM, GRU, RNN-LSTM, RNN-GRU, LSTM-RNN, GRU-RNN, LSTM-GRU, and GRU-LSTM. We present the architecture of the time series forecasting algorithms in Table 2. We limit the hidden layers on each architecture into two. The choice of using two hidden layers is based on the minimal architectural requirement to effectively combine neural network models.

**Table 2:** The architecture of the benchmarked neural network-based time series forecasting algorithms.

Model type	# hidden layers	Architecture			Total parameters
		Type	Output shape	# Param	
RNN	2	Conv1D	(None, 30, 64)	256	16833
		SimpleRNN	(None, 30, 64)	8256	
		SimpleRNN	(None, 64)	8256	
		Dense	(None, 1)	65	
		Lambda	(None, 1)	0	
LSTM	2	Conv1D	(None, 30, 64)	256	66369
		LSTM	(None, 30, 64)	33,024	
		LSTM	(None, 64)	33,024	
		Dense	(None, 1)	65	
		Lambda	(None, 1)	0	
GRU	2	Conv1D	(None, 30, 64)	256	50241
		GRU	(None, 30, 64)	24,960	
		GRU	(None, 64)	24,960	
		Dense	(None, 1)	65	
		Lambda	(None, 1)	0	
RNN-LSTM	2	Conv1D	(None, 30, 64)	256	41601
		SimpleRNN	(None, 30, 64)	8256	
		LSTM	(None, 64)	33,024	
		Dense	(None, 1)	65	
		Lambda	(None, 1)	0	
RNN-GRU	2	Conv1D	(None, 30, 64)	256	33537
		SimpleRNN	(None, 30, 64)	8256	
		GRU	(None, 64)	24,960	
		Dense	(None, 1)	65	
		Lambda	(None, 1)	0	
LSTM-RNN	2	Conv1D	(None, 30, 64)	256	41601
		LSTM	(None, 30, 64)	33,024	
		SimpleRNN	(None, 64)	8256	
		Dense	(None, 1)	65	
		Lambda	(None, 1)	0	
LSTM-GRU	2	Conv1D	(None, 30, 64)	256	58305
		LSTM	(None, 30, 64)	33,024	
		GRU	(None, 64)	24,960	
		Dense	(None, 1)	65	
		Lambda	(None, 1)	0	
GRU-RNN	2	Conv1D	(None, 30, 64)	256	33537
		GRU	(None, 30, 64)	24,960	
		SimpleRNN	(None, 64)	8256	
		Dense	(None, 1)	65	
		Lambda	(None, 1)	0	
GRU-LSTM	2	Conv1D	(None, 30, 64)	256	58305
		GRU	(None, 7, 64)	24,960	
		LSTM	(None, 64)	33,024	
		Dense	(None, 1)	65	
		Lambda	(None, 1)	0	

Furthermore, we set the numbers of parameter to be relatively the same, except RNN and GRU-RNN that need fewer numbers of parameters. GRU-RNN models have fewer parameters because both GRU and vanilla RNN have simpler architectures compared to LSTM, resulting in less complexity and fewer trainable weights [18].

### Model evaluation

To prepare the benchmarking, we need to define the training and test split of each dataset, as described by the last row of Table 1. We evaluate the forecasting performance of each model using three error metrics and computational efficiency metric. The error metrics consist of Mean Absolute Error (MAE), Mean Absolute Percentage Error (MAPE), and Root Mean Square Error (RMSE), defined as follows [20], [21]:

(2.2) $$\text{MAE}=\frac{1}{n}\sum_{i=1}^{n}\left|y_{i}-{\hat{y}}_{i}\right|$$

where $y_{i}$ represents the actual value, ${\hat{y}}_{i}$ represents the predicted value, and n is the number of data points. MAE quantifies the average magnitude of prediction errors in the original units of measurement, providing direct interpretability of model deviation from observed values.

(0) $$\text{MAPE}=\frac{100}{n}\sum_{i=1}^{n}\left|\frac{y_{i}-{\hat{y}}_{i}}{y_{i}}\right|$$

MAPE expresses the error as a percentage of the actual values, offering a scale-independent measure particularly useful for comparing predictions across different magnitudes of the time series data observation.

(1) $$\text{RMSE}=\sqrt{\frac{1}{n}\sum_{i=1}^{n}{\left(y_{i}-{\hat{y}}_{i}\right)}^{2}}$$

RMSE, calculated as the square root of the average squared differences between predicted and actual values, penalizes larger errors more heavily due to its quadratic nature, making it particularly sensitive to outliers in the time series data. Additionally, computation time was measured to assess the practical feasibility of each model for real-time speed predictions. This comprehensive evaluation framework allows for a balanced assessment of both prediction accuracy and computational efficiency, essential factors in the reliability of time series analysis.

### Monte Carlo-based evaluation

We implement MC simulation as a comprehensive benchmarking approach for neural network models. This evaluation framework implements random sampling through multiple iterations of model training, where each iteration begins with a different random weight initialization and performs time series forecasting on the training dataset. The methodology enables robust analysis of model performance across repeated experiments, providing insights into the models’ reliability and stability.

The evaluation protocol consists of 100 independent runs, each comprising 100 training epochs. The resulting performance metrics and predictions are analyzed using a 95 % confidence interval, where we trim 2.5 % from both upper and lower bounds to eliminate extreme outliers. This statistical approach provides a reliable estimation of model parameters and their associated uncertainty ranges. This probabilistic approach adheres to the fundamental principles of Monte Carlo methods, which facilitate the estimation of complex high-dimensional integrals through random sampling processes [22], [23]. By aggregating results across multiple stochastic trials, our framework quantifies prediction uncertainty and parameter sensitivity, offering a more complete characterization of model behavior than single-run evaluations.

Besides model performance evaluation using MAE, MAPE, and RMSE across 100 independent runs on three datasets, statistical comparison was conducted using the Friedman test [24]. The test represents the superior analytical method, especially when evaluating multiple different models across several datasets, as it is a non-parametric alternative to repeated measures ANOVA that tests whether model rankings differ significantly across datasets and iterations [25], [26].

## Benchmarking result and discussion

We present the analysis of our benchmarking results across the datasets, followed by a detailed discussion of the findings. We evaluate the performance of various neural network models using both error metrics and computational efficiency measures.

### Experimental settings

All experiments were conducted using Google Colab’s standard runtime environment with an Intel Xeon CPU and 12.72 GB RAM. The implementation was developed using Python with essential data science and machine learning frameworks. For numerical computing and data structure manipulation, we utilized NumPy and pandas respectively. Data visualization was accomplished through Matplotlib, while the deep learning models were implemented using TensorFlow 2.0. This software stack ensures reproducibility and provides robust tools for time series analysis and neural network implementation.

### Results

We present our experimental results across three distinct datasets, analyzing both the predictive accuracy and computational efficiency of each model. The results are evaluated using MAE, MAPE, RMSE, and computation time metrics, with uncertainty quantified through MC simulation of 100 iterations.

Table 3 shows the Friedman Test results for the nine RNN architectures and Table 4 shows the overall model performance rankings based on Friedman test analysis. The Friedman test revealed no statistically significant differences among the nine RNN architectures ($\chi^{2}=12.593$, df=8, p=.127). Although the test statistic suggested some variation in model performance, the differences were not sufficient to reject the null hypothesis of equal performance at the α=0.05 significance level.

**Table 3:** Performance comparison of RNN architectures using Friedman test analysis across three datasets. Values represent mean ± standard deviation for each metric. Mean ranks indicate relative performance (lower is better). Models are grouped by dataset and ordered by overall performance ranking.

Dataset	Model	MAE ± SD	MAPE ± SD	RMSE ± SD	Mean Rank	Tier
Sunspot Dataset	LSTM-GRU	16.913 ± 0.629	38.637 ± 3.999	23.205 ± 0.827	**2.23**	**Best**
	LSTM-RNN	16.961 ± 0.677	37.277 ± 3.635	23.386 ± 0.966	**3.24**	**Excellent**
	GRU	17.367 ± 1.172	38.569 ± 5.277	23.814 ± 1.449	3.62	Excellent
	GRU-LSTM	17.416 ± 1.119	36.242 ± 3.627	24.091 ± 1.560	4.57	Good
	LSTM	17.176 ± 0.759	38.500 ± 3.654	23.557 ± 1.115	4.67	Good
	RNN-LSTM	17.607 ± 1.074	41.948 ± 5.025	24.165 ± 1.536	4.86	Good
	RNN-GRU	18.321 ± 1.289	42.570 ± 4.278	25.043 ± 1.791	6.16	Below Average
	GRU-RNN	17.533 ± 0.812	43.702 ± 5.989	24.159 ± 1.129	7.09	Poor
	RNN	18.876 ± 1.546	50.941 ± 5.751	25.678 ± 2.197	8.57	Poor
Covid-19 Dataset	LSTM-GRU	0.903 ± 0.122	9.349 ± 0.961	1.149 ± 0.176	**2.23**	**Best**
	LSTM-RNN	0.909 ± 0.102	9.201 ± 0.880	1.156 ± 0.139	**3.24**	**Excellent**
	GRU	0.903 ± 0.122	9.349 ± 0.961	1.149 ± 0.176	3.62	Excellent
	GRU-LSTM	0.909 ± 0.102	9.201 ± 0.880	1.156 ± 0.139	4.57	Good
	LSTM	0.903 ± 0.091	9.036 ± 0.778	1.164 ± 0.129	4.67	Good
	RNN-LSTM	0.956 ± 0.121	10.052 ± 1.099	1.215 ± 0.172	4.86	Good
	RNN-GRU	1.021 ± 0.176	10.996 ± 1.547	1.310 ± 0.252	6.16	Below Average
	GRU-RNN	1.065 ± 0.156	11.331 ± 1.554	1.370 ± 0.218	7.09	Poor
	RNN	1.208 ± 0.230	12.901 ± 1.917	1.592 ± 0.347	8.57	Poor
Oxygen Dataset	LSTM-GRU	3.023 ± 0.386	7.538 ± 0.977	4.100 ± 0.395	**2.23**	**Best**
	LSTM-RNN	2.970 ± 0.229	7.420 ± 0.585	4.041 ± 0.242	**3.24**	**Excellent**
	GRU	3.175 ± 0.396	7.987 ± 1.115	4.204 ± 0.370	3.62	Excellent
	GRU-LSTM	3.261 ± 0.412	8.215 ± 1.195	4.281 ± 0.387	4.57	Good
	LSTM	3.258 ± 0.438	8.152 ± 1.100	4.358 ± 0.482	4.67	Good
	RNN-LSTM	3.099 ± 0.441	7.720 ± 1.122	4.203 ± 0.462	4.86	Good
	RNN-GRU	3.099 ± 0.354	7.740 ± 0.927	4.212 ± 0.349	6.16	Below Average
	GRU-RNN	3.241 ± 0.438	8.141 ± 1.239	4.278 ± 0.431	7.09	Poor
	RNN	3.322 ± 0.566	8.251 ± 1.435	4.455 ± 0.576	8.57	Poor

**Table 4:** Overall model performance rankings based on Friedman test analysis. Rankings are derived from mean ranks across all datasets and evaluation metrics. Statistical analysis: $\chi^{2}=12.593$, df = 8, p=.127 (not significant).

Rank	Architecture	Mean Rank	Performance Tier
**1**	**LSTM-GRU**	**2.230**	**Best**
**2**	**LSTM-RNN**	**3.240**	**Excellent**
**3**	**GRU**	**3.620**	**Excellent**
4	GRU-LSTM	4.570	Good
5	LSTM	4.670	Good
6	RNN-LSTM	4.860	Good
7	RNN-GRU	6.160	Below Average
8	GRU-RNN	7.090	Poor
9	RNN	8.570	Poor

Despite the lack of statistical significance, descriptive analysis revealed performance variations among the architectures. The LSTM-GRU hybrid architecture achieved the lowest mean rank (2.23), while the vanilla RNN showed the highest mean rank (8.57). However, these differences should be interpreted with caution given the non-significant omnibus test result. Hybrid architectures consistently outperformed single-architecture models, with LSTM-GRU achieving the lowest mean rank (2.23).

#### Sunspot dataset

**Fig. 4:**
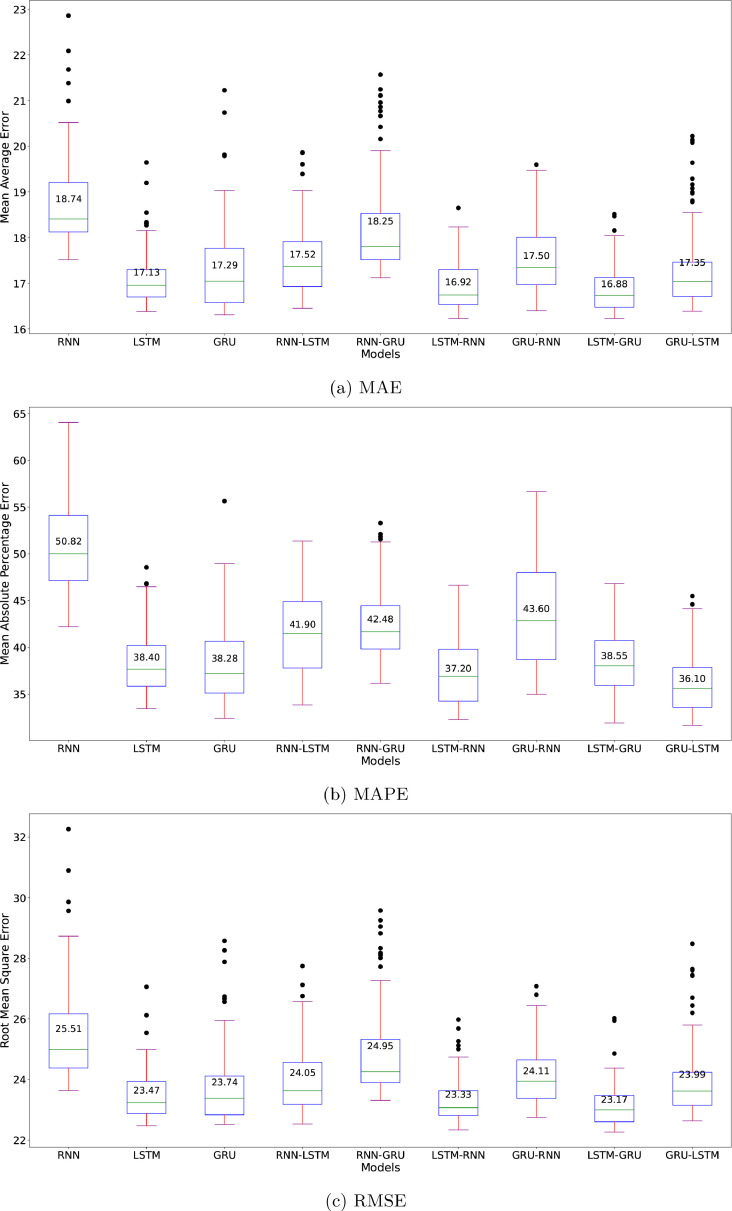
Benchmark of the nine algorithms’ evaluation metrics of Sunspot dataset.

Fig. 4 depicts the performance metrics for the nine algorithms in the sunspot dataset with the MC evaluation. From our comprehensive analysis of MAE, MAPE, and RMSE metrics across different neural network architectures, LSTM-based models consistently demonstrate superior performance and stability. The LSTM-GRU hybrid achieves the lowest median MAE (16.88) with minimal spread, followed by LSTM-RNN with median MAE of 16.92. On the other hand, GRU-LSTM shows the best median MAPE (36.10 %) with the smallest spread, followed by LSTM-RNN with median MAPE of 37.20. For RMSE, the LSTM-GRU architecture outperforms hybrid variants with the lowest median (23.17) and smallest spread, followed by LSTM-RNN and LSTM. This pattern suggests that LSTM-RNN architecture, as the second-best performer, provide robust and reliable predictions for time series forecasting for the sunspot dataset which exhibits cyclical-like patterns.

Analysis of error metrics shows that RNN-based architectures exhibit consistent underperformance patterns. The vanilla RNN model exhibits the highest error rates and largest variances (MAE: 18.74, MAPE: 50.82 %, RMSE: 25.51), highlighting its inherent limitations in capturing long-term dependencies in sunspot time series data. The substantial MAPE value is particularly noteworthy, indicating that RNN predictions deviate by approximately half of the true values. Given the cyclical-like nature of sunspot activity, ranging from near-zero to 400 counts, such large percentage errors significantly impact the model’s reliability. While the error spread remains moderately consistent, suggesting some degree of predictable behavior, the magnitude of these errors underscores RNN’s fundamental challenges in time series forecasting compared to the more sophisticated LSTM and GRU-based architectures.

**Fig. 5:**
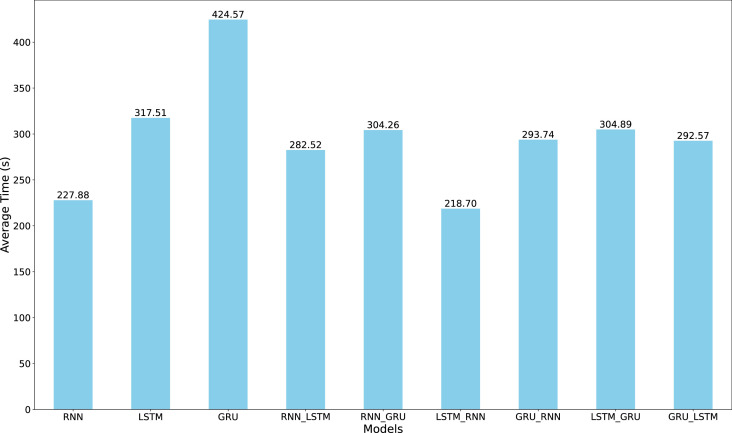
The LSTM-RNN model achieved the lowest mean computation time among the others to train with the sunspot dataset.

The comparative analysis of the sunspot dataset demonstrates that hybrid architectures, particularly LSTM-RNN, outperform single architectures while maintaining computational efficiency. While the vanilla RNN model exhibits higher variability and larger error rates, the LSTM-RNN hybrid achieves a balanced trade-off between prediction accuracy and processing overhead, as evidenced in Fig. 5. The vanilla RNN and some RNN-combined models (RNN-LSTM and LSTM-RNN) achieve relatively shorter training time than the others. Thus, the short training time and better performance of LSTM-RNN over 100 iterations of suggested that, for the sunspot dataset, it has the most reliable performance across all three error metrics without incurring excessive computational costs. This suggests that LSTM-RNN offers a pragmatic solution for sunspot prediction, effectively balancing accuracy and resource utilization.

#### Covid-19 dataset

**Fig. 6:**
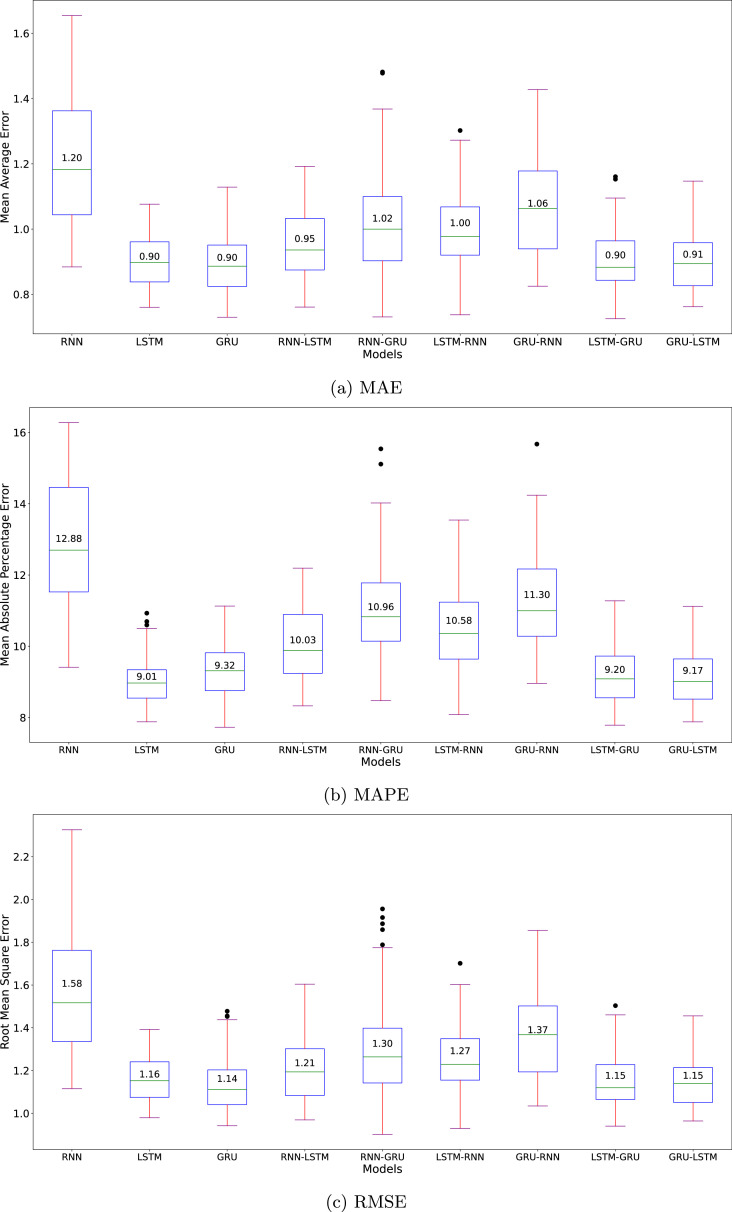
Benchmark of the nine algorithms’ evaluation metrics of Covid-19 dataset.

The performance metrics of the nine algorithms towards the Indonesia Covid-19 daily new cases dataset can be seen in Fig. 6. LSTM, GRU, LSTM-GRU, and GRU-LSTM models achieve median MAEs of 0.90–0.91 where LSTM having the smallest spread. This pattern is also consistent with RMSE, where the medians of those four models span around 1.14 and 1.16. These patterns suggest that the new cases forecasting of those four models have error close to 0.01. On the other hand, the MAPE metrics suggest that the vanilla LSTM significantly outperformed the other models, with close margin on the medians and the smallest spread. Thus, the vanilla LSTM architecture performed relatively the best in this dataset with our MC-based evaluation on the random initialization over 100 iterations.

The MC evaluation of COVID-19 case forecasting, illustrated in Fig. 6, reveals consistent limitations of the RNN architecture, mirroring our findings from the sunspot analysis. The RNN model exhibits notably higher median values across all metrics (MAE: 1.20, MAPE: 12.88 %, RMSE: 1.58) compared to other architectures. Moreover, its substantial spread in error distributions-evidenced by wider box plots and numerous outliers in the metrics-indicates significant prediction instability. This high variability, coupled with larger error magnitudes, suggests that RNN may not be reliable to capture the patterns inherent in Indonesia Covid-19 new daily cases dataset.

**Fig. 7:**
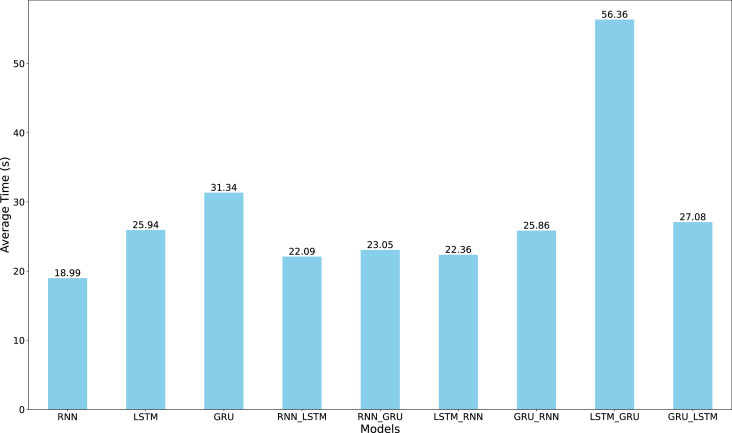
Comparison of the mean training time among the nine algorithms for Covid-19 dataset. The RNN model inherently achieved the shortest time among the others.

Fig. 7 illustrates the mean computation time for training each of the nine architectures on the COVID-19 dataset. While the vanilla RNN demonstrates notably efficient training times, requiring less than 20 seconds per iteration, its computational advantage is overshadowed by its inferior forecasting performance. Among the top-performing models-LSTM, GRU, LSTM-GRU, and GRU-LSTM-the computational requirements vary significantly. Although LSTM-GRU achieves competitive accuracy, its higher computational overhead makes it less practical for COVID-19 case forecasting. Balancing predictive accuracy with computational efficiency, our analysis suggests that single LSTM and GRU-LSTM architectures emerge as the most promising candidates for COVID-19 forecasting applications, offering reliable performance without excessive computational demands.

#### Oxygen dataset

**Fig. 8:**
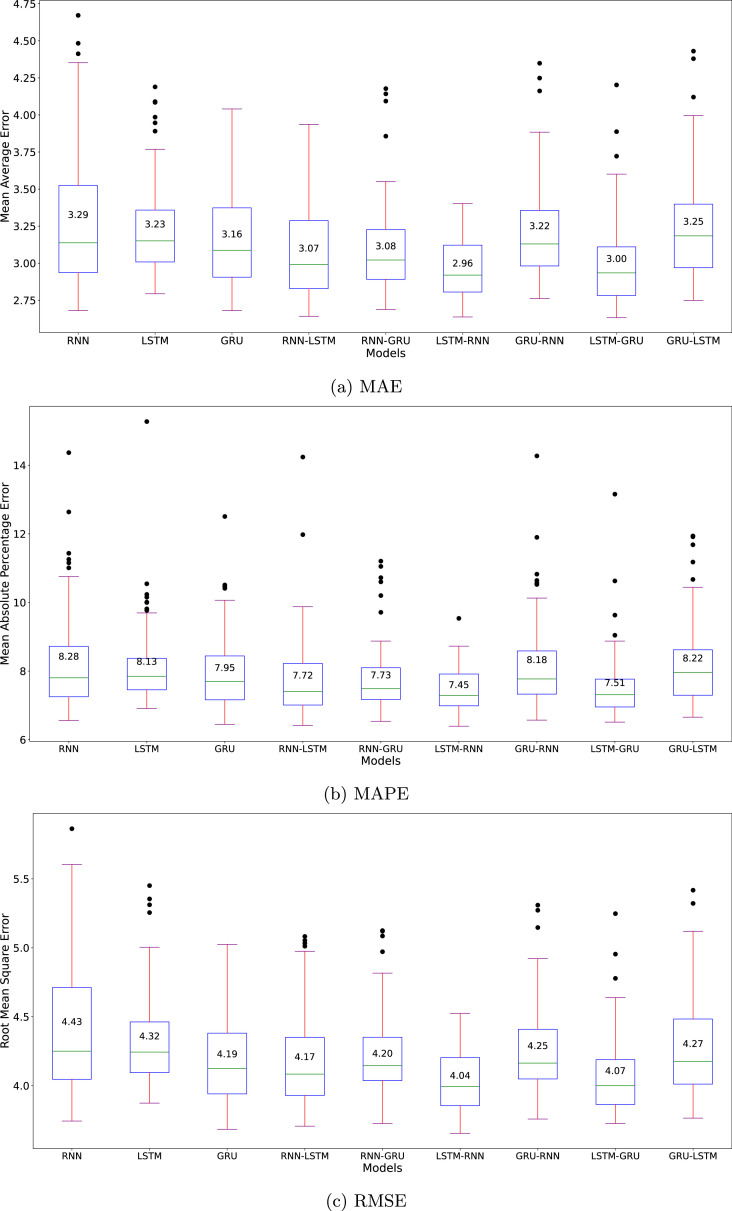
Benchmark of the nine algorithms’ evaluation metrics of Oxygen dataset.

Fig. 8 illustrates the Monte Carlo evaluation results for the Oxygen dataset across all architectures. The LSTM-RNN hybrid demonstrates marginally better performance with the lowest error rates (MAE: 2.96, MAPE: 7.45 %, RMSE: 4.04), followed closely by LSTM-GRU. Both hybrid architectures exhibit not only competitive accuracy but also smaller variances in their predictions. This stability suggests enhanced reliability in handling the dataset’s challenging characteristics, particularly the pronounced fluctuations in the initial readings and the upward trend in dissolved oxygen concentrations observed in the latter portion of the dataset.

The vanilla RNN architecture, while showing only marginally higher median errors (MAE: 3.29, MAPE: 8.28 %, RMSE: 4.43), exhibits notably larger variance across all metrics. This increased spread, particularly visible in the extended whiskers and outliers of the box plots, indicates less consistent prediction reliability. The wider error distribution suggests that RNN’s performance is more susceptible to initial conditions and training variations, making it a less dependable choice for oxygen level forecasting despite its similar median performance to other models.

**Fig. 9:**
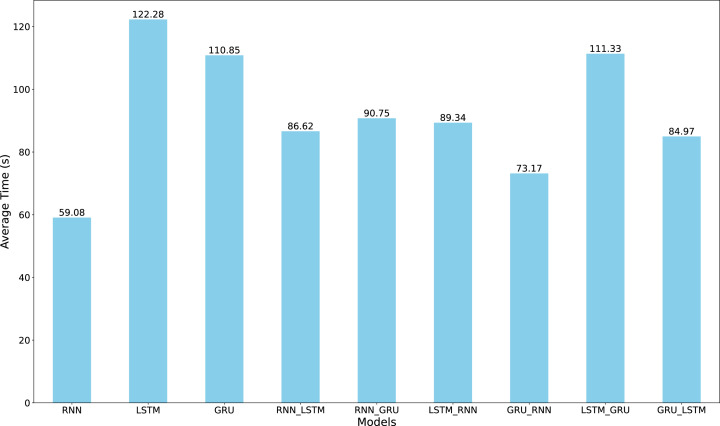
Comparison of the mean training time among the nine algorithms for Oxygen dataset. The RNN model inherently achieved the shortest time among the others.

Fig. 9 presents the mean training time across all architectures, with RNN, GRU-RNN, and GRU-LSTM achieving the fastest training times. While GRU-LSTM requires only 84.97 seconds for training, its marginal computational advantage is offset by slightly higher error metrics compared to LSTM-RNN. The LSTM-RNN architecture, despite requiring 89.34 seconds for training-a mere 5-second difference-demonstrates superior forecasting accuracy across all metrics. This minimal computational overhead suggests that LSTM-RNN offers an optimal balance between prediction accuracy and computational efficiency for dissolved oxygen concentration forecasting, as the slight increase in training time yields meaningful improvements in prediction reliability.

### Discussion

Selecting an optimal neural network architecture for time series forecasting requires careful consideration of both computational efficiency and prediction performance. Our benchmark investigation across three diverse datasets-Sunspot activity, COVID-19 cases, and dissolved oxygen concentration-reveals nuanced differences in model capabilities. Through Monte Carlo evaluation of nine different architectures, including both single and hybrid configurations, we observe using boxplot analysis that while LSTM-RNN demonstrates marginally better performance, the differences are not consistently significant across all datasets. This observation aligns with the “no free lunch” theorem, suggesting that architectural advantages may be dataset-specific rather than universal.

The benchmark reveals that while the vanilla RNN consistently achieves the fastest computation time across all datasets, it demonstrates significant limitations in forecasting accuracy. For the Sunspot dataset, as seen on Table 3, RNN shows the highest error rates and largest standard deviation (MAE: 18.876 ± 1.546, MAPE: 50.941 % ± 5.751 %, RMSE: 25.678 ± 2.197), indicating fundamental limitations in capturing long-term dependencies. Similar patterns emerge in the COVID-19 dataset, where RNN’s performance metrics suggest insufficient capability to model complex epidemiological patterns. Although the differences are less pronounced in the Oxygen dataset (MAE: 3.322 ± 0.566, MAPE: 8.251 % ± 1.435 %, RMSE: 4.455 ± 0.576), RNN still exhibits larger variance, indicating less reliable predictions despite competitive median performance.

Conversely, LSTM-based architectures, particularly in hybrid configurations, demonstrate superior performance. The LSTM-RNN hybrid achieves remarkable accuracy across datasets, with only marginal computational overhead compared to more efficient models. For instance, in the Oxygen dataset, LSTM-RNN requires only 5 seconds of additional training time compared to GRU-LSTM, while delivering better prediction stability. Given these marginal computational trade-offs and consistent performance advantages, LSTM-RNN emerges as a promising choice for time series forecasting applications where prediction reliability is crucial- although simpler architectures such as RNNs offer faster training times.

Refer to Fig. 9, our findings from Oxygen Dataset in line with previous studies such as Shewalkar et al. [27], who advocate for GRU over LSTM based on computational efficiency. In our benchmark, GRU shows superior performance to LSTM. The effectiveness of hybrid architectures, particularly LSTM-GRU, indicates that combining complementary architectural strengths can yield improved forecasting capabilities without excessive computational demands. It is important to note here, although the Friedman test showed no statistically significant differences among the nine RNN architectures ($\chi^{2}=12.593$, df=8, p=.127), descriptive analysis revealed notable performance variations. The LSTM-GRU hybrid architecture achieved the lowest mean rank (2.23), suggesting potential practical advantages despite the lack of statistical significance.

However, several limitations should be considered. While the benchmarked neural network models demonstrate robust handling of noise and outliers in the COVID-19 and Oxygen datasets, the Sunspot dataset’s wider value range presents more significant challenges for accurate prediction. These limitations suggest opportunities for future research, including investigating adaptive architectures that can better handle varying data scales and characteristics. Such studies could enhance our understanding of neural network capabilities across different data distributions and lead to more versatile forecasting solutions.

While this study presents comparative results across multiple neural network architectures using consistent Monte Carlo-based evaluations, formal statistical significance test (e.g., Friedman test) was applied to strengthen comparative conclusion [24], [25], [26]. Using the MAE, MAPE, and RMSE value, the best architecture vary, such as for the Sunspot Dataset, when evaluated using MAPE, the top three algorithms are GRU-LSTM, LSTM-RNN, and LSTM, with MAPE values of 36.242 ± 3.627, 37.277 ± 3.635, and 38.500 ± 3.654, respectively. When evaluated using RMSE, the test indicates that LSTM-GRU, LSTM-RNN, and LSTM demonstrate RMSE values of 23.205 ± 0.827, 23.386 ± 0.966, and 23.557 ± 1.115, respectively. The differences observed between MAPE and RMSE results can be attributed to their fundamental characteristics. MAPE expresses accuracy as a percentage, making it suitable for relative error assessment, whereas RMSE focuses on absolute prediction accuracy and penalizes larger errors more heavily. This suggests that the best-performing model depends on the specific aspect being evaluated. Model robustness is reflected in its consistency across multiple metrics, while domain expertise remains essential for selecting the appropriate evaluation priority.

The Friedman test showed no statistically significant differences among the nine RNN architectures ($\chi^{2}=12.593$, df=8, p=.127). However, this result should be interpreted within the context of the experimental design and practical considerations.

Despite the lack of statistical significance, several observations merit discussion:

• The LSTM-GRU hybrid achieved consistently lower mean ranks (2.23) across all datasets
• Hybrid architectures generally outperformed single-architecture models in descriptive analysis
• The vanilla RNN showed consistently poor performance (mean rank: 8.57)

In terms of practical application, the findings of this study might have promising applicability across various real-world sectors. For example, the proposed approach can be embedded in early warning systems to anticipate events before they occur. In the field of medical monitoring, it may assist in tracking patient conditions and facilitating early preventive actions. Furthermore, in environmental surveillance, the model can be used to monitor changes in climate, air quality, or water resources, supporting proactive environmental management. These applications demonstrate the broader utility of our approach beyond theoretical performance, particularly in scenarios requiring continuous, real-time decision support.

## Conclusions

In this study, nine different neural network architectures, including RNN, LSTM, GRU, and their hybrid configurations, were evaluated using three real-world time series datasets: sunspot activity, Indonesian COVID-19 cases, and dissolved oxygen concentration readings. Model performance was assessed through one hundred iterations of Monte Carlo simulation using multiple evaluation metrics, including mean absolute error (MAE), mean squared error (MSE), root mean squared error (RMSE), and computation time. The Friedman test revealed no statistically significant differences among the nine RNN architectures ($\chi^{2}=12.593$, df=8, p=.127). This non-significant result may be attributed to several factors: first, the limited sample size with only three datasets used for evaluation, and second, performance differences that may have been masked by experimental noise. While statistical significance was not achieved, the consistent performance patterns observed across datasets suggest potential practical advantages for hybrid architectures that warrant further investigation with larger sample sizes or different experimental conditions.

Despite performance variations across individual datasets, our analysis identified several persistent characteristics. The vanilla RNN, although computationally efficient, consistently demonstrated inferior forecasting capabilities across all datasets, exhibiting higher error rates and greater forecast variability. In contrast, LSTM-based architectures, particularly the LSTM-RNN and LSTM-GRU hybrids, consistently exhibited superior performance across diverse datasets, albeit with slightly longer computation times. Specific architectural strengths emerged for different prediction tasks. The LSTM-RNN configuration demonstrated exceptional potential for forecasting sunspot activity and dissolved oxygen concentrations, while the standalone LSTM architecture performed particularly well in COVID-19 case prediction, especially when evaluated using MAE and MAPE metrics. These findings indicate that LSTM-based architectures provide robust performance across various time series characteristics, despite marginal differences in some metrics that align with the “no free lunch” principle.

The study’s limitations primarily stem from the constrained experimental design, including the limited number of datasets and potential masking of performance differences by experimental variability. For future research, we recommend several improvements: increasing the number of datasets to enhance statistical power, implementing alternative statistical approaches such as Bayesian analysis, and focusing on practical significance alongside statistical measures. Additionally, future investigations could explore adaptive neural network architectures that dynamically adjust parameters such as neuron count and activation functions to optimize time series forecasting capabilities across diverse data distributions. Such adaptive approaches may provide more robust solutions for real-world applications where data characteristics vary significantly.

For practical implications, while the statistical analysis did not reveal significant differences among architectures, the consistent performance patterns observed provide valuable guidance for practitioners. LSTM-based hybrid architectures, particularly LSTM-RNN and LSTM-GRU configurations, demonstrate reliable performance across multiple domains and should be considered as primary candidates for time series forecasting applications. However, the choice of architecture should also consider computational constraints and specific application requirements, as the vanilla RNN may still be suitable for scenarios where computational efficiency outweighs marginal performance gains.

## Supplementary material

https://github.com/arianayunita/performance-analysis-of-hybrid-models-

## CRediT authorship contribution statement

**Ariana Yunita:** Conceptualization, Validation, Formal analysis, Supervision. **MHD Iqbal Pratama:** Visualization, Software, Data curation. **Muhammad Zaki Almuzakki:** Methodology. **Hani Ramadhan:** Visualization, Writing – review & editing. **Emelia Akashah P. Akhir:** Resources. **Andi Besse Firdausiah Mansur:** Writing – review & editing. **Ahmad Hoirul Basori:** Writing – review & editing.

## Declaration of competing interest

The authors declare that they have no known competing financial interests or personal relationships.

## Footnotes

2 SIDC and Quandl. Database from SIDC – Solar Influences Data Analysis Center – the solar physics research department of the Royal Observatory of Belgium. SIDC (https://www.sidc.be/)- downloaded from https://www.kaggle.com/datasets/robervalt/sunspots

3 The Covid-19 is downloaded from https://www.kaggle.com/datasets/hendratno/covid19-indonesia
